# Collecting Paediatric Health-Related Quality of Life Data: Assessing the Feasibility and Acceptability of the Australian Paediatric Multi-Instrument Comparison (P-MIC) Study

**DOI:** 10.3390/children10101604

**Published:** 2023-09-26

**Authors:** Renee Jones, Rachel O’Loughlin, Xiuqin Xiong, Mina Bahrampour, Kristy McGregor, Shilana Yip, Nancy Devlin, Harriet Hiscock, Brendan Mulhern, Kim Dalziel, on behalf of the Quality of Life in Kids: Key Evidence to Strengthen Decisions in Australia (QUOKKA) Project Team

**Affiliations:** 1 Health Economics Unit, Centre for Health Policy, The University of Melbourne, Melbourne, VIC 3010, Australia; reneej1@student.unimelb.edu.au (R.J.); oloughlin.r@unimelb.edu.au (R.O.); xiuqinx@student.unimelb.edu.au (X.X.); nancy.devlin@unimelb.edu.au (N.D.); 2 Health Services and Economics, Murdoch Children’s Research Institute, Melbourne, VIC 3052, Australiaharriet.hiscock@rch.org.au (H.H.); 3 Health Services Research Unit, Royal Children’s Hospital, Melbourne, VIC 3052, Australia; 4 Centre for Health Economics Research and Evaluation, University of Technology Sydney, Ultimo, NSW 2007, Australia; mina.bahrampour@uts.edu.au (M.B.); brendan.mulhern@uts.edu.au (B.M.); 5 Department of Paediatrics, The University of Melbourne, Melbourne, VIC 3010, Australia

**Keywords:** paediatric, quality of life, cost–benefit analysis, patient reported outcome measures, feasibility, acceptability, quality

## Abstract

Background: Collecting data using paediatric health-related quality of life (HRQoL) instruments is complex, and there is a paucity of evidence regarding the comparative performance of paediatric HRQoL instruments. The Australian Paediatric Multi-Instrument Comparison (P-MIC) study was conducted to address this paucity of evidence. This study aims to understand the (1) feasibility of collecting data using paediatric HRQoL instruments in a research setting and (2) acceptability and feasibility for children and their caregivers to complete common paediatric HRQoL instruments using data from the Australian P-MIC study. Methods: Data were from children aged 5–18 years from the Australian P-MIC study. Demographics, cost and time for data collection, dropout rates, and inconsistent responses were used to assess Aim 1. Participant-reported difficulty and completion time were used to assess Aim 2. Subgroup analyses included child age, report type (self/proxy), sample recruitment pathway (hospital/online), and online panel sample type (general population/condition groups). Results: Overall, 5945 P-MIC participants aged 5–18 years completed an initial survey, of these, 2346 also completed the follow-up survey (39.5% response rate). Compared with online panel recruitment, hospital recruitment was more costly and time-consuming and had higher follow-up completion (33.5% versus 80.4%) (Aim 1). Data were of similar good quality (based on inconsistent responses) for both recruitment pathways (Aim 1). Participants completed each instrument in <3 min, on average, and >70% reported each instrument as easy to complete (Aim 2). Conclusions: The Australian P-MIC study was able to collect good-quality data using both online panel and hospital recruitment pathways. All instruments were acceptable and feasible to children and their caregivers.

## 1. Introduction

Paediatric health-related quality of life (HRQoL) instruments are standardised instruments that capture a child’s perception of their own HRQoL (child self-report) or a caregiver’s perception of their child’s HRQoL (proxy report) [1,2]. In recent decades, there has been increasing recognition of the importance of the child’s voice in paediatric healthcare delivery and decision-making [1,3]. Consequently, the availability and use of paediatric HRQoL instruments have rapidly expanded, resulting in a wide range of instruments to choose from [4,5,6].

HRQoL instruments have commonly been used in clinical trials and are increasingly used for other applications such as routine clinical care, population health research, and clinical registries [3]. Economic evaluation utilises HRQoL information accompanied by preference weights to generate quality-adjusted life year (QALY) estimates, a metric combining HRQoL and survival impacts [5]. In economic evaluation, these QALY estimates are used as the unit of measurement to understand the health improvement of a pharmaceutical or health intervention. They can then be compared to the cost of a pharmaceutical or health intervention to inform a cost–utility analysis [5]. HRQoL instruments are also used in routine clinical care as patient-reported outcome measures (PROMs). A PROM is an outcome measure, such as an HRQoL instrument, that is completed by a patient to inform healthcare providers about health outcomes from their perspective [7]. PROMs can assist in the identification of new health problems, clinical decision-making about treatments, and clinician–patient communication, although evidence is largely focused on adult patients [7,8]. Additionally, HRQoL instruments are used in population studies and clinical registries, allowing for large-scale assessment of population quality of life to inform policy and evaluation of health system performance [9]. To be useful in these real-world applications, it is crucial that HRQoL instruments are acceptable to users, feasible to collect and implement, and accurate in measuring HRQoL issues of relevance to the target population.

Despite the wide range of paediatric HRQoL instruments now available, there are evidence gaps regarding the comparative performance of these instruments [10,11,12]. Most evidence is restricted to single instruments and or populations, precluding comparisons across instruments [10,11,12]. To fill these evidence gaps, we have undertaken a study involving the collection of common paediatric HRQoL instruments head-to-head at two time points across a large sample of children, known as the Australian Paediatric Multi-Instrument Comparison (P-MIC) study [13,14]. Consensus-based Standards for the Selection of Health Measurement Instruments (COSMIN) guidelines outline that understanding the quality of methods used to obtain data about HRQoL instrument performance is vital to ensure the appropriate and transparent interpretation of conclusions [15]. As the P-MIC study data are intended to be used to generate large amounts of evidence regarding paediatric HRQoL instrument performance, it is essential to understand the quality of the data collected in the study (i.e., the data reported by a participant is largely consistent within the survey and reflects a genuine response). Furthermore, participants contributing to the development of HRQoL evidence can be recruited using different sample recruitment pathways, such as online panels or clinical samples. Understanding the differences in feasibility when collecting data using HRQoL instruments with these different recruitment pathways is of international relevance in informing similar research in the future and further developing the evidence base for paediatric HRQoL instruments. A 2022 American study reported on the feasibility of collecting online data using online panel recruitment [16]. The 2022 study highlighted that a key consideration for the feasibility of collecting data using online panel recruitment pathways was data quality and wasteful recruitment, necessitating the removal of 32% of participants for failing data quality eligibility criteria [16]. Despite this, no evidence is available on the feasibility, including data quality, of collecting data using the same survey with different sample recruitment pathways (i.e., hospital recruitment pathway versus online panel recruitment pathway). 

The acceptability of children and their caregivers to complete paediatric HRQoL instruments has previously been captured largely based on data missingness [10]. However, other aspects of acceptability, such as users’ own perspectives on the difficulty or ease of completing instruments, are lacking. Similarly, the feasibility of completing HRQoL instruments is often evaluated by assessing time to complete; however, only a few studies across different populations have presented such data for paediatric HRQoL instruments [17,18,19]. Furthermore, the existing evidence on the acceptability and feasibility of paediatric HRQoL instruments is focused on single instruments and or populations (e.g., specific conditions and age groups), precluding head-to-head comparisons of instrument acceptability and feasibility across a range of contexts [10,11].

The Australian P-MIC study was conducted to address current gaps in the literature regarding the comparative performance of paediatric HRQoL instruments. As part of the generation of the Australian P-MIC data, this study aims to (1) understand the feasibility of collecting data using paediatric HRQoL instruments in a research setting and (2) understand the acceptability and feasibility for children and their caregivers to complete common generic paediatric HRQoL instruments. This study also aims to understand how Aims 1 and 2 compare by child age (5–12 years versus 13–18 years), instrument report type (proxy versus self-report), sample recruitment pathway (hospital versus online panel), and online panel sample type (general population versus specific health condition groups).

## 2. Methods

### 2.1. Study Design and Participants

The P-MIC study prospectively collected demographic information concurrent with multiple generic paediatric HRQoL instruments from Australian children and their caregivers [13,14]. P-MIC study data from children aged 5 to 18 years from Data Cut 2, dated 10 August 2022, were used, which include approximately 94% of the total planned P-MIC participants [13]. Although the P-MIC study also includes responses from children as young as two years old, children aged two to four years were not included in this analysis as HRQoL instruments are largely experimental in this age group except Pediatric Quality of Life Inventory Generic Core 4.0 (PedsQL).

The P-MIC study received ethics approval from the Royal Children’s Hospital Human Research Ethics Committee on 21 March 2021 (HREC/71872/RCHM2021). All participants, including caregivers and children (if relevant), provided informed consent prior to completing the survey. The study was designed to recruit adult caregivers of children aged 2–18 years; hence, caregivers were asked to provide informed consent after completing eligibility questions. After the adult caregiver consented, they were asked to complete demographic questions, including the child’s age. If they had a child aged seven years or above, they were asked if the child was currently able to complete some questions about their health and wellbeing. If the caregiver said their child was able to complete some questions about their health and wellbeing, the caregiver was asked to hand the survey over to the child, who was then asked to provide informed consent. 

Participants were recruited between June 2021 and August 2022. In the P-MIC study, a range of different participant samples were recruited to enable the assessment of instrument performance in different child populations. Specifically, participants were recruited into three samples: Sample (1): children with or without health conditions were consecutively recruited via a large tertiary paediatric hospital based in Victoria, Australia (hereon referred to as hospital recruitment pathway), Sample (2): Australian general population children recruited via an online panel (available in all states and territories across Australia), Pureprofile Australia, and Sample (3): children from nine condition-specific groups (attention-deficit/hyperactivity disorder (ADHD), anxiety and/or depression, autism spectrum disorder (ASD), asthma, eating disorder, epilepsy, recurrent abdominal pain, sleep problems, and tooth problems) recruited via the same online panel as above. Recruitment for Samples 2 and 3 is hereon referred to as the online panel recruitment pathway.

Child age quotas were set during the recruitment of children to the online panel general population sample (Sample 2) to ensure an even distribution across child ages. The purpose of an even distribution across child ages was to ensure enough of a sample size to enable the investigation of instrument performance for all child ages rather than a representative distribution. Child age quotas could not be set during the recruitment of children with one of the nine health conditions from the online panel (Sample 3) due to being harder to recruit. However, quotas for the number of children recruited to each health condition were set with the aim of having approximately 400 children recruited to each health condition. The aim of this quota was to achieve a large enough sample size for each condition to enable the investigation of instrument performance in each health condition. Furthermore, the recruitment of children via the online panel pathway (Sample 2 and 3) was hierarchical, where participants were recruited to the health condition with the lowest prevalence first; if they had no health condition, they were recruited to the general population sample. For rarer conditions in Sample 3 (epilepsy and eating disorders), the online panel sample sizes were increased with targeted recruitment through tertiary paediatric hospital departments and consumer organisations associated with the condition. For the recruitment of participants to Sample 1 (hospital recruitment pathway), strategies included online advertisements with a link to the study placed on all telehealth appointments, research assistants approaching families in waiting rooms of a range of hospital departments, emails or letters sent to eligible patients, and poster advertisements in high-traffic areas of the hospital. Through the recruitment of participants to these different samples, researchers were able to explore instrument performance that may be generalisable to the Australian population whilst also allowing for exploration of performance in key sub-groups.

Eligibility criteria were set *a priori* by the project team and finalised following the completion of a soft launch involving the recruitment of approximately 250 respondents via the online panel recruitment pathway (Samples 2 and 3) to test online panel recruitment. Participants were eligible if they (1) were the caregiver of a child aged 2–18 years, (2) met the screening criteria for the relevant condition if recruited into the condition-specific sample (Sample 3) (see Section 5 of the Technical Methods Paper for further details), (3) they completed at least the initial survey (including consent), (4) were not a duplicate respondent, and (5) met minimum sample quality eligibility criteria (see Section 12 of the Technical Methods Paper for further details) [13].

### 2.2. Data Collection

Participants were invited to complete two surveys online: an initial survey and a follow-up survey. All surveys were completed using REDCap, an online survey platform. Most participants were invited to complete the follow-up survey four weeks after completing the initial survey; however, a random subset of participants from the online panel general population sample (Sample 2) was invited to complete the follow-up survey two days after the initial survey to allow for a test–retest reliability assessment. All paediatric HRQoL instruments were self-completed online by either the child (child self-report) or their caregiver (proxy report) (i.e., no instruments were interviewer-administered). The paediatric HRQoL instruments were completed by the child (child self-report) if they were aged seven years or older and their caregiver reported that they were able to complete questions about their health and wellbeing. Where this was not possible or where children were younger than seven years of age, the HRQoL instruments were completed by the caregiver (proxy report). Where instruments were proxy-reported, they were asked to report the child’s HRQoL from their perspective. Further information on the P-MIC study methodology, including details of participant recruitment, is reported in a Technical Methods Paper [13].

Although most data used in this study were obtained from the participant surveys collected online using REDCap, some data relating to Aim 1, namely, the time and cost of the P-MIC study data collection, were collected from study management records. 

### 2.3. Instruments and Analysis

The following common generic paediatric HRQoL instruments were collected in both the initial and follow-up surveys and included in the analysis: PedsQL; EQ-5D Youth (EQ-5D-Y) 3L and 5L; Child Health Utility 9D (CHU9D); Assessment of Quality of Life 6D (AQol-6D) adolescent; Health Utilities Index Mark 2 and 3 (HUI2/3); and Patient-Reported Outcomes Measurement Information System 25 paediatric profile v2 (PROMIS-25). All instruments aim to capture aspects of health and wellbeing that are common to most children; hence, they are referred to as ‘generic’. Table 1 provides a description of the features of each instrument, including the number of items, recall period, outcome scale, outcome levels, and domains assessed. All participants completed the PedsQL, EQ-5D-Y-3L, EQ-5D-Y-5L, and CHU9D. To minimise the responder burden in the sample recruited via the hospital recruitment pathway (Sample 1), these participants did not complete the HUI 2/3, PROMIS-25, or AQoL-6D. To minimise responder burden in the samples recruited via the online panel recruitment pathway (Samples 2 and 3), participants were randomised to receive only one of HUI 2/3, PROMIS-25, or AQoL-6D. The order participants received the PedsQL, EQ-5D-Y-3L, EQ-5D-Y-5L, and CHU9D was randomised to minimise order effects or effects of responder burden. Additionally, there was always a different instrument completed between the EQ-5D-Y-3L and EQ-5D-Y-5L given their similarity. The HUI 2/3, PROMIS-25, and AQoL-6D were received after the other generic HRQoL instruments. To reduce the survey burden, the EQ visual analogue scale (EQ VAS) was only attached to the EQ-5D-Y-3L and not the EQ-5D-Y-5L; hence, participants only needed to complete this once. Condition-specific instruments were also included for participants in the condition-specific group sample (Sample 3); however, they are not included in this analysis.

### 2.4. Statistical Analysis

Analyses were completed using Stata Version 17 (Statacorp, College Station, TX, USA). Where appropriate, subgroup analyses were completed using initial survey data for the following pre-specified sub-groups: child age (5–12 versus 13–18 years), report type (proxy- versus self-report), recruitment type (hospital (Sample 1) versus online panel (Samples 2 and 3)), and online panel sample type (general population (Sample 2) versus condition-specific groups (Sample 3)). For further information on these categorisations, including justifications, please see the psychometric analysis guide available in the P-MIC Technical Methods Paper [13].

#### 2.4.1. Aim 1: Understand the Feasibility of Collecting Data Using Paediatric HRQoL Instruments in a Research Setting

The *types of respondents and response rates achieved* with the P-MIC study methodology were assessed descriptively in the following ways: sociodemographic factors including child, family, and caregiver characteristics [13] and follow-up survey completion rates—calculated as the number of participants who completed the follow-up survey divided by the number of participants who completed the initial survey. These data were collected using participant surveys. Pearson’s chi-squared test, a test used to assess if there are significant differences in the outcome of a categorical variable between specified groups, was also completed to assess if sociodemographic factors differed between sub-groups. Results with a *p*-value < 0.05 were considered significant.

The *time and cost of P-MIC study data collection* were also assessed descriptively in the following ways: time taken to obtain ethics—calculated as the time in months from the date the ethics application was started (the date of the first meeting to start planning the ethics application) to the date the final ethics approval was received, the number of ethics amendments is also described; time taken to recruit—calculated as the time in months from the first participant recruited to the last participant recruited for data cut 2; and the average cost per participant—calculated as the estimated cost of recruiting participants, including research assistant costs, online panel company costs, instrument costs (HUI2/3 and the Strengths and Difficulties Questionnaire (SDQ)), and participant reimbursement costs, divided by the number of participants. All other instruments were free for use in the non-commercial P-MIC study or covered by institutional licenses. Survey development and project management costs were not included. Costs are presented in Australian Dollars (AUD) in the year 2021. Data used to assess the time and cost of P-MIC study data collection were collected from study management records.

The *quality of data collected* in the P-MIC study was also descriptively assessed in the following ways: survey total time to complete and inconsistent responses for similar items. These data were collected from participant surveys. The time to complete the whole initial and follow-up surveys was calculated by adding together the time it took to complete each instrument and section of the survey (including demographics, consent, and other non-HRQoL instruments, if relevant). If participants left the instrument open on their electronic device, this ‘break-time’ would be included; hence, times were top-coded at 3600 s (60 min) and 2400 s (40 min) for the initial and follow-up surveys, respectively, as times above this were not considered to be reasonable. To assess the extremity of survey completion times, the total time to complete the survey was categorised as short (lowest 10%), average (middle 80%), and long (highest 10%). Inconsistent responses for similar items were assessed by evaluating the proportion of participants who responded extremely inconsistently (+/− three levels) and very inconsistently (+/− two levels) to similar items. The most similar items were chosen for assessment; these included EQ-5D-Y-5L (pain)/CHU9D (pain) and EQ-5D-Y-5L (looking after self)/CHU9D (daily routine). 

#### 2.4.2. Aim 2: Understand the Acceptability and Feasibility for Children and Their Caregivers to Complete Common Generic Paediatric HRQoL Instruments

The acceptability and feasibility for children and their caregivers to complete common generic paediatric HRQoL instruments were evaluated by assessing self-reported difficulty completing each instrument and the time to complete each instrument. These data were collected using participant surveys. Participants were asked to rate the difficulty of each instrument directly after completing the instrument on a 5-point scale from 1 ‘very difficult’ to 5 ‘very easy’. Differences in difficulty across instruments and sub-groups were assessed using Pearson’s chi-squared test, with a *p*-value < 0.05 considered significant. The time to complete each instrument was collected using automated functions in REDCap, the online survey platform. This automated time to complete function was based on the number of seconds the instrument was open before the participant clicked on the next instrument. Participants could not click back. If participants left the instrument open on their electronic device, this ‘break-time’ would be included. Hence, times were top-coded at 600 s (10 min), as times above this were not considered to be reasonable. 

## 3. Results

**Figure 1:**
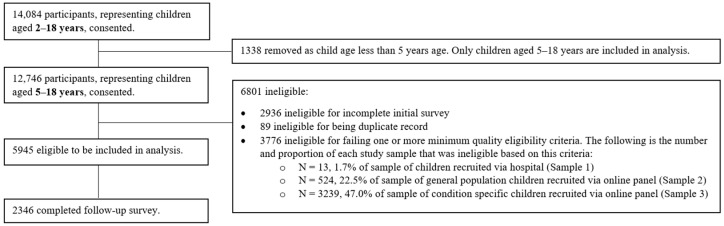
Participant Flow Chart.

Figure 1 summarises the participant flow. A total of 14,084 participants, representing children aged 2–18 years, consented to take part in the P-MIC study. Of those, 1338 were removed, as these represented children aged 2–4 years, which is out of the scope of this analysis; 2936 were ineligible for not completing the initial survey; 89 were ineligible for being a duplicate record; and 3776 were ineligible for failing one or more minimum quality eligibility criteria, leaving 5945 eligible children aged 5–18 years and their caregivers included in analysis. A higher proportion of participants recruited via the online panel pathway were ineligible for failing one or more minimum quality eligibility criteria compared with the proportion of participants recruited via the hospital pathway. 

### 3.1. Aim 1: Understand the Feasibility of Collecting Data Using Paediatric HRQoL Instruments in a Research Setting

The sample characteristics of the 5945 eligible participants are presented in Table 2. A total of 759 were children recruited via the hospital pathway (Sample 1), 1531 were general population children recruited via an online panel (Sample 2), and 3655 were children from one of nine condition-specific groups recruited primarily via an online panel (Sample 3). An even spread of ages and genders was achieved for both online panels and the hospital setting. Children recruited via the hospital pathway were not required to be a patient of the hospital; however, 623 (82.1%) reported they were a patient. Of the children recruited via the hospital pathway (Sample 1), 79.7% had a chronic condition or disability lasting more than six months compared with 48.8% of the online panel condition group sample (Sample 3). Compared with online panel samples (Samples 2 and 3), participants recruited via the hospital pathway (Sample 1) had lower representation from single-parent households (29.7% versus 21.9%) and a higher proportion of caregivers with a bachelor’s degree or above (34.9% versus 46.3%). The chi-squared analysis comparing these differences resulted in a *p*-value of <0.001.

Of the 5945 participants who completed the initial survey, 2346 (39.1%) completed the follow-up survey. Follow-up survey completion rates were much higher in participants recruited via the hospital pathway (80.4%) compared with participants recruited via online panels (33.5%). Additionally, follow-up survey completion rates were higher in participants from the online panel general population sample (39.2%) compared with participants from the online panel condition groups sample (31.1%). 

The participants recruited via the hospital cost more per participant (AUD 79.70) compared with participants recruited via the online panel (AUD 22.60). Among online panel participants, children from the general population sample cost less per participant (AUD 11.60) compared with the condition-specific group sample (AUD 27.70), due to the cost being based on the prevalence of the condition. Additionally, the sample recruited via the hospital took longer to recruit (12 months) compared with the online panel sample (6 months). The time from the first ethics planning meeting to obtaining initial ethics approval was 7.5 months. Eight ethics amendments were required throughout the course of this study.

Table 3 summarises the number and percentage of participants reporting inconsistent responses for similar items. Very few participants reported extremely inconsistent responses (+/− 3 levels) for similar items, with 34 (0.6%) participants and 90 (1.5%) participants reporting extremely inconsistent responses (+/− 3 levels) on the EQ-5D-Y-5L (pain)/CHU9D (pain) and EQ-5D-Y-5L (looking after self)/CHU9D (daily routine), respectively, in the initial survey. The median time to complete across short (lowest 10%), average (middle 80%), and long (highest 10%) groups was generally consistent with expectations for survey length after accounting for differing survey length across sub-groups (See Supplementary Table S1). 

### 3.2. Aim 2: Understand the Acceptability and Feasibility for Children and Their Caregivers to Complete Common Generic Paediatric HRQoL Instruments

Over 70% of participants found each instrument either ‘somewhat’ or ‘very’ easy to complete in the initial survey: EQ-5D-Y-5L (n = 4594, 77.3%), CHU9D (n = 4520, 76.0%), EQ-5D-Y-3L (inc EQ VAS) (n = 4426, 74.5%), AQoL-6D (n = 1123, 73.7%), HUI 2/3 (n = 1260, 72.9%), PedsQL (n = 4301, 72.4%), and PROMIS-25 (n = 1247, 72.2%). See Supplementary Figure S1a–g for a summary of self-reported difficulty for each instrument by sub-group. Compared with the other instruments, the EQ-5D-Y-5L (not including the EQ VAS) had the highest proportion of participants reporting it as being easy to complete. Additionally, all chi-squared analyses comparing the participant-reported difficulty of the EQ-5D-Y-5L (not including the EQ VAS) to each other instrument resulted in a *p*-value of <0.001. For all instruments except the AQoL-6D, participants (either proxies or children self-reporting) in the younger child age group (5–12-year-olds) reported significantly more ease (*p*-value < 0.001) in completing instruments compared with the older child age group (13–18-year-olds). For all instruments, participants from the online panel general population sample reported significantly more ease (*p*-value < 0.001) in completing instruments compared with the online panel condition-specific sample.

Table 4 summarises the time taken in seconds to complete each instrument by sub-groups. The median time to complete varied across instruments, with the shortest being the EQ-5D-Y-5L (29.0 s to complete 5 items, not including the EQ VAS) and the longest being the AQoL-6D (147.2 s to complete 20 items). In the follow-up survey, participants reported significantly greater ease of completion (*p*-value < 0.001) and completed instruments in less time (*p*-value < 0.001) compared with the initial survey. Participants recruited via the online panel recruitment pathway had significantly quicker instrument completion times (*p*-value < 0.001) for all instruments compared to those recruited via the hospital pathway. Additionally, participants from the online panel general population sample had quicker instrument completion times for all instruments compared with participants from the online panel condition-specific sample; however, this was only significant (*p*-value < 0.05) for the EQ-5D-Y-3L (inc the EQ VAS), EQ-5D-Y-5L (not inc the EQ VAS), CHU9D, and AQoL-6D.

## 4. Discussion

We have outlined (1) the feasibility of collecting data using paediatric HRQoL instruments in a research setting, (2) the acceptability and feasibility for children and their caregivers to complete commonly used paediatric HRQoL instruments, and (3) how these compare by child age, report type, sample recruitment pathway, and online panel sample type. In terms of the feasibility of collecting data using paediatric HRQoL instruments in a research setting, the results demonstrate that recruiting samples of children via the hospital pathway compared with the online panel pathway was more costly (AUD 79.7 versus AUD 22.6 per participant) and more time consuming (12- versus 6-months). However, the sample of children recruited via the hospital pathway compared with those recruited via the online panel pathway had a higher follow-up survey completion rate (80.4% versus 33.5%) and were more chronically unwell (79.7% versus 37.3%). The quality procedures put in place for the P-MIC study and the commitment to eligibility criteria ensured good-quality data from eligible participants across all samples. All instruments had strong acceptability and feasibility to children and their caregivers, with each instrument having more than 70% of participants reporting it as ‘somewhat’ or ‘very’ easy to complete and participants completing each instrument in less than 3 min, on average. The EQ-5D-Y-5L instrument, not including the EQ VAS, was the quickest instrument to complete, and participants reported it as the easiest of all instruments; this finding was consistent across all sub-groups.

The P-MIC study is the first of its kind to collect common paediatric HRQoL instruments concurrently across a wide range of child ages, conditions, and settings. It allows for a direct comparison of paediatric HRQoL instruments within the same large sample of children, which is currently missing from the literature [10,11]. The P-MIC data also include children and families from a range of socioeconomic groups, geographic locations, and cultural groups. The P-MIC study was designed with strong sample quality assessment procedures, resulting in high-quality data from samples recruited via the online panel and hospital recruitment pathways, enabling robust conclusions to be drawn from the data [15]. The methods used to collect P-MIC data and ensure data quality can inform future international work collecting HRQoL data electronically using multiple pathways. 

This study has several limitations. Firstly, due to survey logistics, it was required that participants answer all questions; hence, we were not able to determine which instrument items resulted in missing data, which is a possible proxy for relevance or acceptability to children and caregivers and is a limitation of this study. However, this has been explored in previous research [10,11,20,32]. Secondly, due to the way in which participants were recruited (hierarchical for the online panel recruitment pathway) and the way in which the data were collected (online survey), the generalisability of results may be limited, which is discussed in more detail below. Finally, a limitation of this study is that the EQ VAS was only attached to the EQ-5D-Y-3L and not the EQ-5D-Y-5L; hence, differences in time to complete and participant-reported ease in completing the EQ-5D-Y-3L and EQ-5D-Y-5L instruments cannot be disentangled from this. 

Compared to the Longitudinal Study of Australian Children (LSAC) population normative data, the general population sample recruited via the online panel pathway (Sample 2) in this P-MIC study had a very similar sociodemographic composition; however, it did have a lower proportion of children with special healthcare needs (17.3% vs. 7.6%) [30]. This difference is likely because of the hierarchical nature of the online panel recruitment pathway, where children were first screened for one of the nine common condition groups (Sample 3) and, only if they did not meet the screening for the condition groups, were they recruited to the general population sample (Sample 2) [13]. It is acknowledged that other recruitment approaches to data collection could have been used and resulted in slightly different characteristics; however, a hierarchical approach was used to ensure that the more difficult-to-reach condition group sample targets were met first. Given that the online panel general population sample (Sample 2) in this study includes a more healthy population of children compared with the Australian population norm, the ability to generalise results will be limited. However, as the sociodemographic composition was similar to the Australian population norm, this sample may be well placed to form a healthy reference group for future analyses assessing the psychometric performance of instruments, such as known group validity analyses.

Participants in the P-MIC study completed instruments faster when compared with completion times available in the literature. The mean time for participants in the P-MIC study to complete the EQ-5D-Y-3L was 46.7 s, whilst completion times for the EQ-5D-Y-3L in studies conducted in South Africa and the United Kingdom (UK) range between 78 and 157 s [17,18,19]. The mean time taken for participants in the P-MIC study to complete the CHU9D was 56.3 s, whilst completion times for the CHU9D in a study conducted in the UK was 148 s [19]. The mean time taken for participants in the P-MIC study to complete the PedsQL was 96.7 s, whilst completion times for the PedsQL in a study conducted in South Africa was 162 s [17]. These differences may be due to the completion mode (i.e., paper versus online or self-reported versus interviewer-administered), study population, or that P-MIC participants complete serval instruments in one sitting, which may cause a learning effect. Furthermore, except for AQoL-6D [33], participants in the P-MIC study completed instruments faster than the completion times estimated by instrument developers [34,35,36]. For example, the PedsQL administration guidelines note that the estimated time to complete is 5 min [36], whilst participants in the P-MIC study completed the PedsQL in a median time of 1.6 min. It is unknown where the instrument developer data comes from that informs these estimates. Estimates provided by instrument developers often do not distinguish between administration modes, sample complexity, and setting; hence, the quicker completion times noted in this study may be due to the online data collection mode. However, instrument developers may also be conservatively estimating time to complete, and future instrument users could consider that these time estimates may be an overestimation if administered online. Researchers collecting these instruments in future should report the time to complete to enable further comparisons. 

Faster completion times were reported for all instruments in participants recruited via the online panel recruitment pathway compared with the hospital recruitment pathway. This is likely due to online panel participants being more familiar with completing surveys and hence, being quicker. Additionally, it may be due to the setting in which participants receive the invitation to complete the survey, as they may then go on to complete the survey in that same setting. Online panel participants are likely to receive the invitation to their email address and can complete the survey when it suits them and in their ideal environment (i.e., on a computer at home). Participants recruited via the hospital pathway are likely to receive the invitation to complete the survey when they are in contact with the hospital, which may be a more stressful time for them to complete the survey and may involve them completing the survey on their mobile phones. Faster completion times and greater ease of completion were reported for all instruments in participants from the general population sample compared with participants from the condition group samples. The faster completion and greater ease of completing in the general population compared with the condition groups may be that the general population sample does not need to so carefully consider which level to choose, as they are more likely to select the lowest severity/frequency outcome level for each item. Consequently, researchers intending to use paediatric HRQoL instruments in the future should consider their intended patient population and how familiar they may be with completing online surveys or how much they may need to choose between different outcome levels of an item. This may then have implications for additional resources required to assist participants. Additionally, although EQ-5D-Y-5L was faster and participants reported it as easier to complete compared with the EQ-5D-Y-3L, this may be because the 3L also included the EQ VAS, whereas the 5L did not. 

Due to the nature of the P-MIC study design, we were unable to assess the feasibility of collecting HRQoL instruments in other common settings, such as clinical care. However, participants completed all instruments more quickly in the follow-up survey, indicating a learning effect and improved acceptability when repeated. This is an indicator of potential acceptability and feasibility, where repeated outcomes are collected such as in routine clinical care or clinical trials. Additionally, if instruments are collected online in a clinical setting in the future, time to complete and patient difficulty or ease to complete is likely to be comparable to participants from this study who were recruited via a paediatric hospital (Sample 1).

Although this study found that collecting data using paediatric HRQoL instruments was feasible via both the hospital and online panel recruitment pathways, each has advantages and disadvantages. Recruitment via the hospital pathway was expensive and time-consuming, however, it was less wasteful as it recruited more eligible participants (only 1.7% of children failed minimum quality eligibility criteria), included participants who had a higher follow-up survey response rate, and included children who were more chronically unwell compared with those recruited via the online panel pathway. Comparatively, for the online panel pathway, 22.5% of the general population children (Sample 2) and 47% of the children with health conditions (Sample 3) failed the minimum quality eligibility criteria. Recruitment via the online panel pathway was more wasteful, and researchers should ensure they include a stipulation in their contract with the online panel company that they will not pay for recruited participants who fail minimum quality eligibility criteria. This was also found in a 2022 American study collecting online data for a general population via an online panel recruitment pathway, which reported that 39% of people recruited failed minimum quality eligibility criteria [16]. Differences in the proportion failing minimum quality eligibility criteria between the P-MIC study and the American study may be due to different country settings, different online panel companies, and the different minimum quality eligibility criteria. Another study published in 2022 examined the data quality of several online recruitment platforms (Amazon Mechanical Turk, CloudResearch, and Prolific) and panels (Qualtrics and Dynata) and found large variations in data quality across platforms and panels [37]. Although this provides a guide for researchers conducting online research in some settings, further research is needed that includes a wider array of research panels and platforms.

Another consideration when collecting data via the online panel recruitment pathway is that sample quotas for low prevalence conditions–for example in our study, the eating disorder and epilepsy samples-were not able to be filled by the online panel company. The results highlight that when recruiting through an online panel sample, researchers may need to over-sample the number of participants expected to obtain a sufficient number of eligible responses or may need to recruit some hard-to-reach samples via other means. This is an important consideration in the future collection of online self-report and valuation data. 

A strategy of the current study that enabled more detailed eligibility and quality checking was the inclusion of a follow-up survey that included additional eligibility validation questions. If this were a single time point study, these ineligible responses would not have been identified. The quality procedures in the P-MIC emphasise the importance of clear eligibility criteria and quality checking, particularly when collecting data using online panels. The data quality procedures established in this study are transparently reported in this study, with further detail available in the Technical Methods Paper, and can be used as a reference for future studies collecting HRQoL data using online panels [13].

## 5. Conclusions

This study tested the feasibility of collecting paediatric HRQoL instruments in a research setting, providing information about the time, cost, and types of participants recruited via different recruitment pathways. Each recruitment pathway had unique benefits—the online panel pathway was cheap and quick, and the hospital pathway recruited participants who had a higher follow-up response rate and who were more chronically unwell. Having a combination of participants recruited via both pathways is recommended for future similar research. Although the P-MIC study was able to obtain good-quality data, researchers should not underestimate how much work is required to ensure adequate quality using strict eligibility criteria when collecting data from online panel participants. This is particularly important as these data often form an important evidence base for health economics and outcomes research. Increased transparency regarding quality eligibility procedures for future studies collecting data from participants who are recruited using online panels is required. 

This study also tested the acceptability and feasibility of administering common paediatric HRQoL instruments to children and their caregivers by child age, child health status, and report type. Evidence suggests that despite some instruments, such as the EQ-5D-Y-5L (not including the EQ VAS) being marginally easier or quicker to complete than others, all were considered feasible and acceptable to children and their caregivers. This finding was consistent across sub-groups. Considering all instruments were feasible and acceptable, future instrument users may wish to make a decision regarding instrument choice based on other aspects of an instrument’s performance.

Future research should assess the feasibility and acceptability of collecting data using paediatric HRQoL instruments in other countries and contexts, such as clinical care. Additionally, future research should assess aspects of instrument performance beyond acceptability and feasibility, such as psychometric properties. 

## Figures and Tables

**Table 1:** Summary of characteristics of generic paediatric HRQoL instruments included in the analysis.

Instrument	Number Items	Recall Period	Outcome Scale	Outcome Levels	Domains Assessed
PedsQL generic core 4.0 [20]	23 items (5–18 years)	Past month	Frequency	5-point scale	Physical functioning, emotional functioning, social functioning, and school functioning.
EQ-5D-Y-3L [21]	5 items	Today	Severity	3-point scale; and EQ VAS, which is a global health measure on a 0–100 sliding scale	Mobility, looking after self, doing usual activities, having pain or discomfort, and feeling worried, sad, or unhappy.
EQ-5D-Y-5L [21]	5 items	Today	Severity	5-point scale	Mobility, looking after self, doing usual activities, having pain or discomfort, and feeling worried, sad, or unhappy.
CHU9D [22,23]	9 items	Today	Severity	5-point scale	Worry, sadness, pain, tiredness, annoyance, school, sleep, daily routine, and activities.
AQoL-6D Adolescent [24,25]	20 items	Past week	Severity	4- to 6-point scale	Independent living, mental health, coping, relationships, pain, and senses.
HUI 2/3 [26,27,28]	15 items	Usual	Severity	4- to 6-point scale	Vision, hearing, speech, ambulation, dexterity, emotion, cognition, and pain.
PROMIS-25 Paediatric Profile v2 [29]	25 items	Past week	Severity (5 items) and frequency (20 items)	5-point scale; except for the pain item, which is on a scale from 0–10	Depressive symptoms, anxiety, physical function–mobility, pain interference, fatigue, and peer relationships.

**Table 2:** Participant characteristics by data collection type, and online panel sample type.

Participant Characteristic	N (% Completed Initial Survey) or Mean (sd)					Australian Population Norm ^a^
	Total Sample	Sample Recruitment Pathway		Online Panel Sample Type		
		Hospital *	Online Panel	General Population **	Condition Specific ***	
*Sample characteristics*						
Completed initial survey, n (%)	5945 (100)	759 (100)	5186 (100)	1531 (100)	3655 (100)	n/a
Completed follow-up survey, n (%)	2346 (39.5)	610 (80.4)	1736 (33.5)	600 (39.2)	1136 (31.1)	n/a
General population 2-day follow-up survey completed of n = 237 allocated, n (% of allocated)	n/a	n/a	n/a	169 (71.3)	n/a	n/a
General population 4-week follow-up survey completed of n = 1361 allocated, n (% of allocated)	n/a	n/a	n/a	431 (31.7)	n/a	n/a
Online panel condition groups (Sample 3)	3655 (61.5)	n/a	n/a	n/a	n/a	n/a
Asthma	487 (8.2)	n/a	n/a	n/a	n/a	n/a
Attention deficit hyperactivity disorder (ADHD)	492 (8.3)	n/a	n/a	n/a	n/a	n/a
Autism spectrum disorder (ASD)	510 (8.6)	n/a	n/a	n/a	n/a	n/a
Anxiety or depression	480 (8.1)	n/a	n/a	n/a	n/a	n/a
Eating disorder	186 (3.1)	n/a	n/a	n/a	n/a	n/a
Epilepsy	272 (4.6)	n/a	n/a	n/a	n/a	n/a
Tooth problems	490 (8.2)	n/a	n/a	n/a	n/a	n/a
Sleep problems	346 (5.8)	n/a	n/a	n/a	n/a	n/a
Recurrent abdominal pain	392 (6.6)	n/a	n/a	n/a	n/a	n/a
HRQoL instrument report type initial survey proxy report, n (%)	2083 (35.0)	306 (40.3)	1777 (34.3)	536 (35.0)	1241 (34.0)	n/a
HRQoL instrument report type follow-up survey proxy report, n (%) of those who have completed follow-up survey)	975 (41.6)	289 (47.4)	686 (39.5)	249 (41.5)	437 (38.5)	n/a
Completed core HRQoL instruments (PedsQL, CHU9D, EQ-5D-Y-3L, EQ-5D-Y-5L)	5945 (100)	759 (100)	5186 (100)	1531 (100)	3655 (100)	n/a
Completed AQoL-6D	1523 (25.6)	n/a	1523 (29.4)	499	1024	n/a
Completed HUI 2/3	1728 (29.1)	n/a	1728 (33.3)	522	1206	n/a
Completed PROMIS-25	1730 (29.1)	n/a	1730 (33.3)	510	1220	n/a
*Study Child characteristics*						
Child age, mean (sd)	10.9 (3.9)	10.6 (3.8)	10.9 (3.9)	11.2 (4.0)	10.8 (3.9)	n/a
Child gender—female, n (%)	2737 (46.0)	333 (43.9)	2404 (46.4)	738 (48.2)	1666 (45.6)	48.7%
Child of Aboriginal and/or Torres Strait Islander origin—yes, n (%)	379 (6.4)	22 (2.9)	357 (6.9)	51 (3.3)	306 (8.4)	3.7%
Child speaks language other than English spoken at home—yes, n (%)	513 (8.6)	92 (12.1)	421 (8.1)	201 (13.1)	220 (6.0)	13.1%
Child has chronic health condition or disability (lasting at least 6 months), n (%)	2537 (42.7)	605 (79.7)	1932 (37.3)	150 (9.8)	1782 (48.8)	n/a
Special healthcare need ^b^—yes, n (%)	2583 (43.5)	572 (75.4)	2011 (38.8)	117 (7.6)	1894 (51.8)	17.3%
*Caregiver and family characteristics*						
Caregiver age, mean (sd)	40.8 (8.5)	42.6 (7.2)	40.5 (8.7)	42.5 (9.1)	39.7 (8.3)	41.1 (mean)
Caregiver highest education level—bachelor’s degree or above, n (%)	2161 (36.4)	351 (46.3)	1810 (34.9)	611 (39.9)	1199 (32.8)	28.9%
Household income AUD 2000 or more per week (AUD 104,000 or more per year), n (%)	1977 (33.3)	274 (36.1)	1703 (32.8)	589 (38.5)	1114 (30.5)	47.9%
Single parent household, n (%)	1679 (28.7)	163 (21.9)	1516 (29.7)	352 (23.3)	1164 (32.3)	17.8%
Remoteness (based on postcode)—major cities, n (%)	4254 (71.6)	568 (74.8)	3686 (71.1)	1150 (75.1)	2536 (69.4)	66.4%

Abbreviations: AQoL-6D—Assessment of Quality of Life; CHU9D—Child Health Utility; EQ-5D-Y—EQ-5D Youth; HUI2/3—Health Utilities Index Mark 2/3; n/a—not applicable; PedsQL—Paediatric Quality of Life Inventory; PROMIS-25—Patient-Reported Outcome Measurement Information System 25. ^a^ Australian normative data obtained from the Longitudinal Study of Australian Children (LSAC) study calculated using LSAC combined seven waves of data (excluding the 0–1-year-old) from two cohort-adjusted population weights [30]. ^b^ Special healthcare need is defined as per the Children with Special Health Care Needs (CSHCN) screener [31]. * Sample 1 (recruited via the hospital pathway), ** Sample 2 (general population sample recruited via the online panel pathway), *** Sample 3 (condition-specific samples recruited via the online panel pathway).

**Table 3:** Quality of HRQoL data for eligible participants (time to complete the whole survey, inconsistent responses, and internal consistency) by data collection type, and online panel sample type.

Quality Variable	N (%) or Median (IQR)					
	Survey		Sample Recruitment Pathway		Online Panel Sample Type	
	Initial	Follow-Up	Hospital *	Online Panel	General Population **	Condition Specific ***
*Extremely inconsistent response (+/− three levels) for similar items*, *n(%)*						
EQ-5D-Y-5L (pain)/CHU9D (pain)	34 (0.6)	52 (2.2)	6 (0.8)	28 (0.5)	7 (0.5)	21 (0.6)
EQ-5D-Y-5L (looking after self)/CHU9D (daily routine)	90 (1.5)	73 (3.1)	20 (2.6)	70 (1.4)	4 (0.3)	66 (1.8)
*Very inconsistent response (+/− two levels) for similar items*, *n(%)*						
EQ-5D-Y-5L (pain)/CHU9D (pain)	189 (3.2)	95 (4.1)	40 (5.3)	149 (2.9)	20 (1.3)	129 (3.5)
EQ-5D-Y-5L (looking after self)/CHU9D (daily routine)	407 (6.9)	161 (6.9)	67 (8.8)	340 (6.6)	31 (2.0)	309 (8.5)

Abbreviations: CHU9D—Child Health Utility; EQ-5D-Y—EQ-5D Youth. Note: excludes participants ineligible for failing minimum quality eligibility criteria and duplicate respondents. * Sample 1 (recruited via the hospital pathway), ** Sample 2 (general population sample recruited via the online pane pathway), *** Sample 3 (condition-specific samples recruited via the online panel pathway).

**Table 4:** Time to complete instrument by survey, child age, report type, sample recruitment pathway, and sample type.

Time to Complete Instrument in Seconds, Median (IQR)										
Instrument	Survey		Child Age		Report Type		Sample Recruitment Pathway		Online Panel Sample Type	
	Initial	Follow-Up	5–12 years	13–18 years	Self-Report	Proxy Report	Hospital *	Online Panel	General Population **	Condition Specific ***
PedsQL (23 items)	96.7 (73.8, 133.5)	90.3 (68.6, 123.4)	96.5 (73.1, 135.8)	97.1 (75.1, 129.5)	96.2 (73.3, 137.6)	97.5 (74.8, 127.1)	125.6 (94.7, 186.4)	93.6 (71.9, 126.5)	90.9 (68.8, 126.7)	94.9 (73.3, 126.3)
EQ-5D-Y-3L (including EQ VAS) (6 items)	46.7 (34.4, 65.8)	43.0 (31.1, 60.9)	46.6 (35.1, 66.6)	46.7 (35.1, 64.9)	46.3 (34.1, 66.2)	47.5 (35.3, 65.3)	58.9 (43.2, 88.7)	45.5 (33.5, 62.9)	42.4 (30.9, 59.1)	46.5 (34.6, 64.3)
EQ-5D-Y-5L (no EQ VAS) (5 items)	29.0 (20.9, 41.8)	27.7 (19.8, 40.8)	29.1 (20.8, 42.4)	28.7 (21.0, 41.2)	29.1 (20.9, 42.4)	28.9 (20.9, 40.4)	36.6 (26.0, 55.4)	28.1 (20.5, 40.1)	25.3 (18.3, 36.3)	29.4 (21.5, 41.4)
CHU9D (9 items)	56.3 (41.5, 79.3)	52.0 (38.4, 75.4)	56.3 (41.1, 80.5)	56.5 (42.5, 77.8)	56.2 (41.4, 80.8)	56.6 (41.8, 77.2)	72.7 (53.1, 101.5)	54.1 (40.3, 75.5)	50.1 (37.1, 72.6)	56.0 (42.0, 76.4)
AQoL-6D (20 items)	147.2 (107.0, 208.2)	132.9 (96.8, 190.6)	146 (102.9, 205.1)	151.6 (111.4, 215.1)	141.6 (101.8, 201.9)	157.9 (115.7, 215.5)	n/a	n/a	135.9 (99.1, 183.7)	152.5 (111.3, 217.8)
HUI 2/3 (15 items)	112.4 (75.5, 167.2)	94.1 (63.5, 144.8)	109.5 (73.8, 163.7)	117.7 (79.2, 173.5)	111.9 (75.7, 163.5)	113.6 (75.0, 171.8)	n/a	n/a	96.5 (68.3, 147.4)	120.0 (81.2, 173.1)
PROMIS-25 (25 items)	98.7 (74, 133.2)	93.3 (70.4, 133.6)	96.8 (72.0, 131.4)	100.7 (77.1, 134.4)	94.5 (71.2, 128.8)	106.1 (80.6, 140.8)	n/a	n/a	94.8 (69.8, 128.1)	100.1 (75.8, 135.3)

Abbreviations: AQoL-6D—Assessment of Quality of Life; CHU9D—Child Health Utility; EQ-5D-Y—EQ-5D Youth; HUI2/3—Health Utilities Index Mark 2/3; n/a—not applicable; PedsQL—Paediatric Quality of Life Inventory; PROMIS-25—Patient-Reported Outcome Measurement Information System 25; VAS—visual analogue scale. * Sample 1 (recruited via the hospital pathway), ** Sample 2 (general population sample recruited via the online panel pathway), *** Sample 3 (condition-specific samples recruited via the online panel pathway).

## Data Availability

The datasets generated during and/or analysed during the current study are available from the corresponding author upon reasonable request.

## Supplementary Materials

The following supporting information can be downloaded at: https://www.mdpi.com/article/10.3390/children10101604/s1, Table S1: Time to complete whole survey in seconds by sample recruitment pathway, and sample type; Figure S1a: PedsQL self-reported difficulty by child age, report type, sample recruitment pathway, and online panel sample type; Figure S1b: EQ-5D-Y-3L (including EQ VAS) self-reported difficulty by child age, report type, sample recruitment pathway, and online panel sample type; Figure S1c: EQ-5D-Y-5L (not including EQ VAS) self-reported difficulty by child age, report type, sample recruitment pathway, and online panel sample type; Figure S1d: CHU9D self-reported difficulty by child age, report type, sample recruitment pathway, and online panel sample type; Figure S1e: AQoL-6D self-reported difficulty by child age, report type, and online panel sample type; Figure S1f: HUI2/3 self-reported difficulty by child age, report type, and online panel sample type. Figure S1g: PROMIS-25 self-reported difficulty by child age, report type, and online panel sample type.
