# Supplementary material for: Collecting Paediatric Health-Related Quality of Life Data: Assessing the Feasibility and Acceptability of the Australian Paediatric Multi-Instrument Comparison (P-MIC) Study
Source: Children (Basel). 2023 Sep 26;10(10):1604. doi: 10.3390/children10101604 (PMC10605580; doi:10.3390/children10101604)
Supplement: Supplementary file 1 [file children-10-01604-s001.zip › children-2592789-supplementary.pdf]

SUPPLEMENTARY MATERIALS

Table of Contents for Supplementary Materials

Supplementary Table S1. Time to complete the whole survey in seconds by sample recruitment pathway and sample type. 1
Figure S1a. PedsQL self-reported difficulty by child age, report type, sample recruitment pathway, and online panel sample type. 2
Figure S1b. EQ-5D-Y-3L self-reported difficulty by child age, report type, sample recruitment pathway, and online panel sample type. 3
Figure S1c. EQ-5D-Y-5L self-reported difficulty by child age, report type, sample recruitment pathway, and online panel sample type. 4
Figure S1d. CHU9D self-reported difficulty by child age, report type, sample recruitment pathway, and online panel sample type. 5
Figure S1e. AQoL-6D self-reported difficulty by child age, report type, and online panel sample type. 6
Figure S1f. HUI2/3 self-reported difficulty by child age, report type, and online panel sample type. 7
Figure S1g. PROMIS-25 self-reported difficulty by child age, report type, and online panel sample type. 8

Supplementary Table S1. Time to complete the whole survey in seconds by sample recruitment pathway, and sample type.

Quality variable	Median (IQR)					
	Survey		Sample recruitment pathway		Online panel sample type	
	Initial	Follow-up	Hospital*	Online panel	General population **	Condition-specific ***
**Time to complete whole survey (seconds)**						
Short (lowest 10%)	339.6 (299.8, 367.8)	168.0 (147.6, 183.0)	343.2 (312.7, 373.0)	339.4 (298.9, 367.3)	338.1 (295.4, 365.0)	340.6 (299.8, 369.1)
Average (middle 80%)	660.7 (534.0, 850.9)	329.4 (263.5, 428.5)	652.2 (534.0, 822.0)	661.5 (534.0, 852.9)	612.8 (499.0, 778.9)	685.2 (551.0, 873.5)
Long (highest 10%)	1,993.9 (1,584.9, 3,251.7)	929.5 (772.7, 1,523.8)	3,009.1 (1,946.9, 3,600)	1,859.4 (1,552.8, 2,670.7)	1,905.9 (1,513.6, 2,849.3)	1,845.9 (1,554.0, 2,656.5)

Sample 1 (recruited via hospital), ** Sample 2 (general population sample recruited via online panel), * Sample 3 (condition-specific samples recruited via online panel)*

Figure S1a. PedsQL self-reported difficulty by child age, report type, sample recruitment pathway, and online panel sample type.

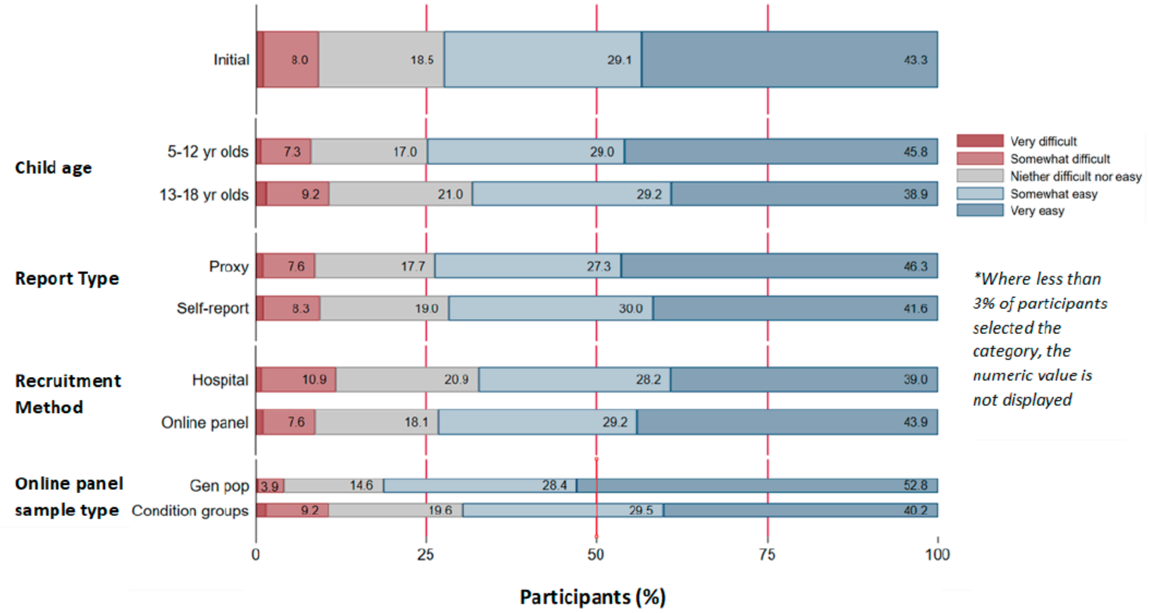

Abbreviations: Gen pop, general population; Initial, initial survey responses.

Figure S1b. EQ-5D-Y-3L (including EQ VAS) self-reported difficulty by child age, report type, sample recruitment pathway, and online panel sample type.

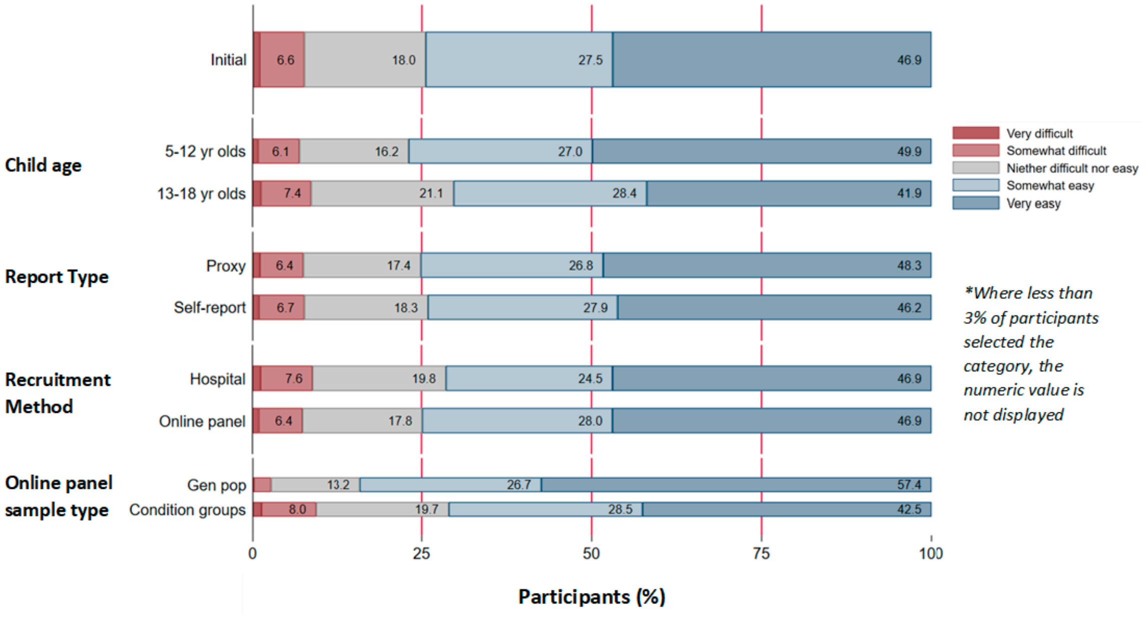

Abbreviations: Gen pop, general population; Initial, initial survey responses.

Figure S1c. EQ-5D-Y-5L (not including EQ VAS) self-reported difficulty by child age, report type, sample recruitment pathway, and online panel sample type.

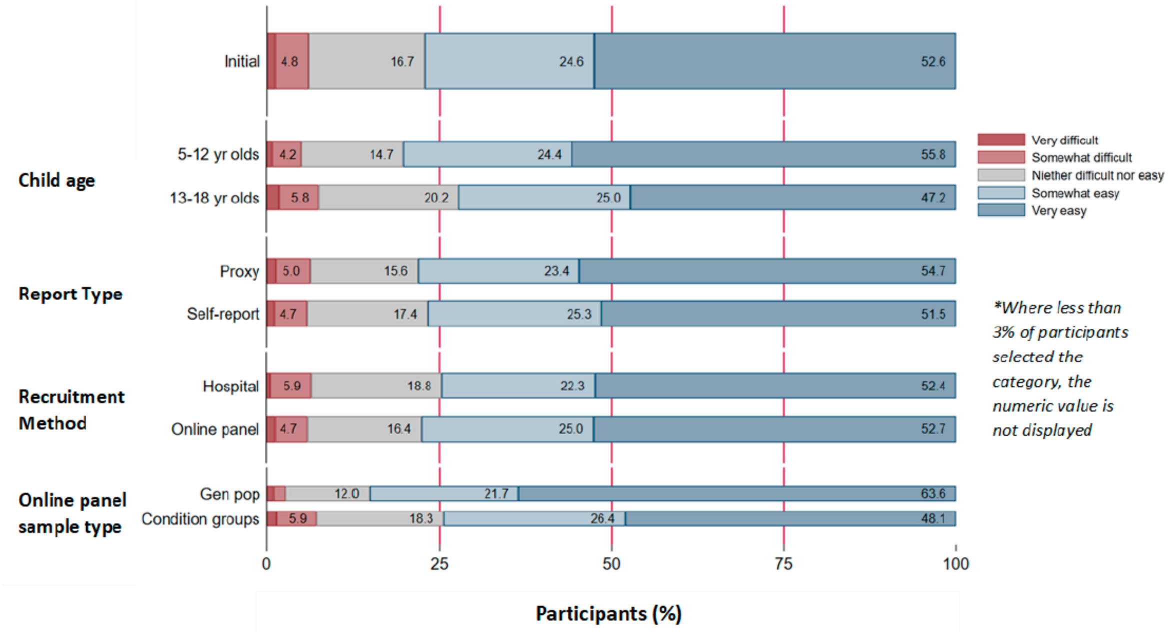

Abbreviations: Gen pop, general population; Initial, initial survey responses.

Figure S1d. CHU9D self-reported difficulty by child age, report type, sample recruitment pathway, and online panel sample type.

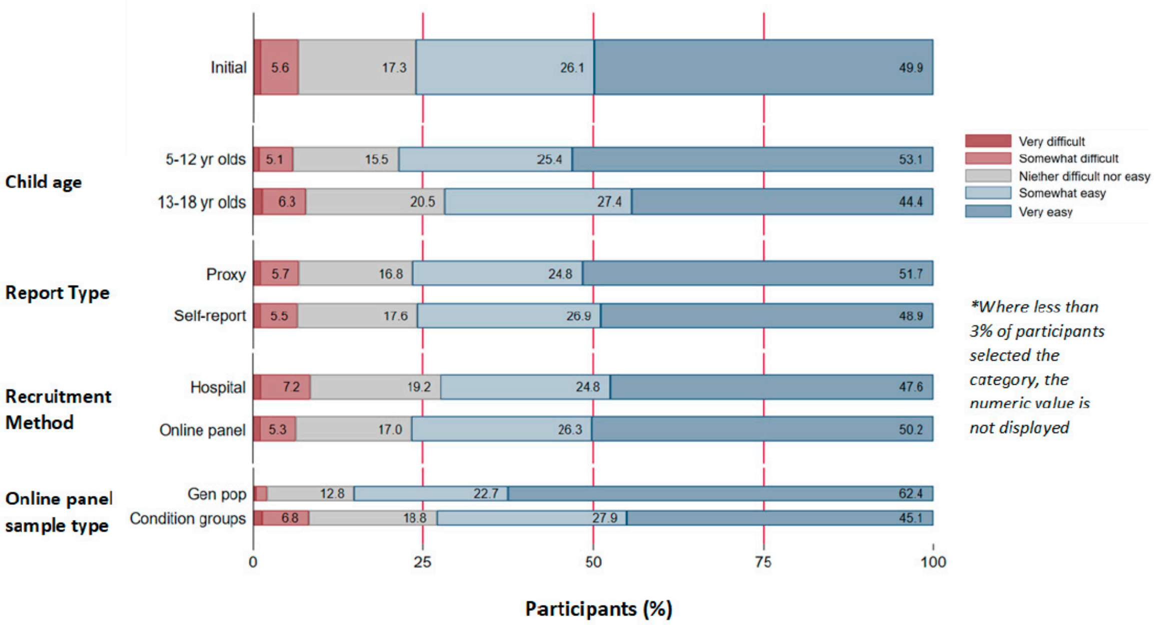

Abbreviations: Gen pop, general population; Initial, initial survey responses.

Figure S1e. AQoL-6D self-reported difficulty by child age, report type, and online panel sample type.

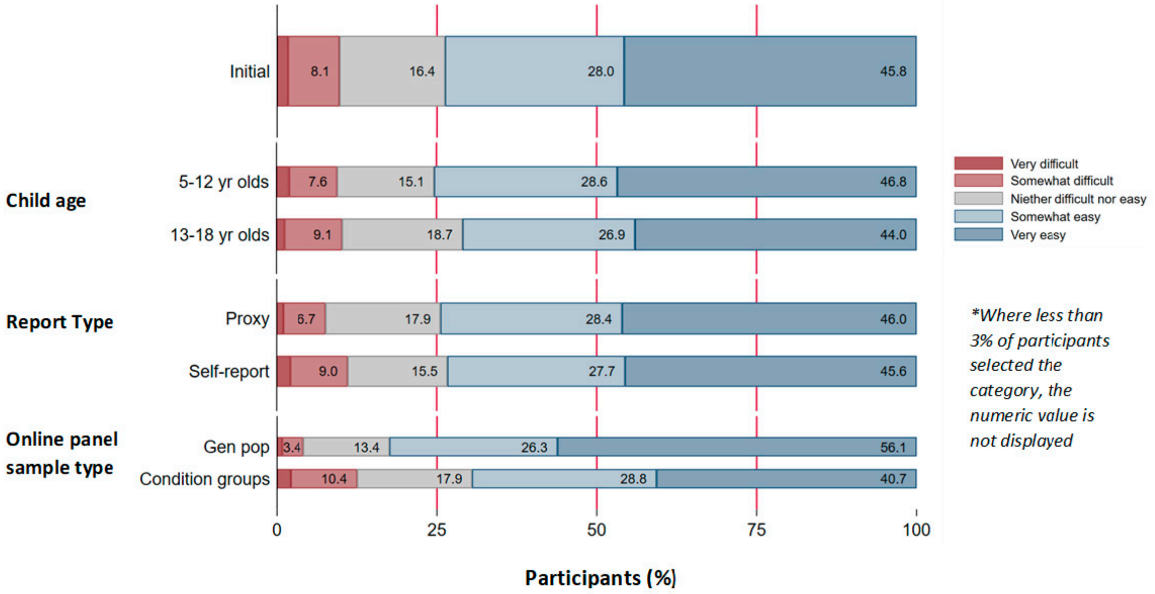

Abbreviations: Gen pop, general population; Initial, initial survey responses.

Figure S1f. HUI2/3 self-reported difficulty by child age, report type, and online panel sample type.

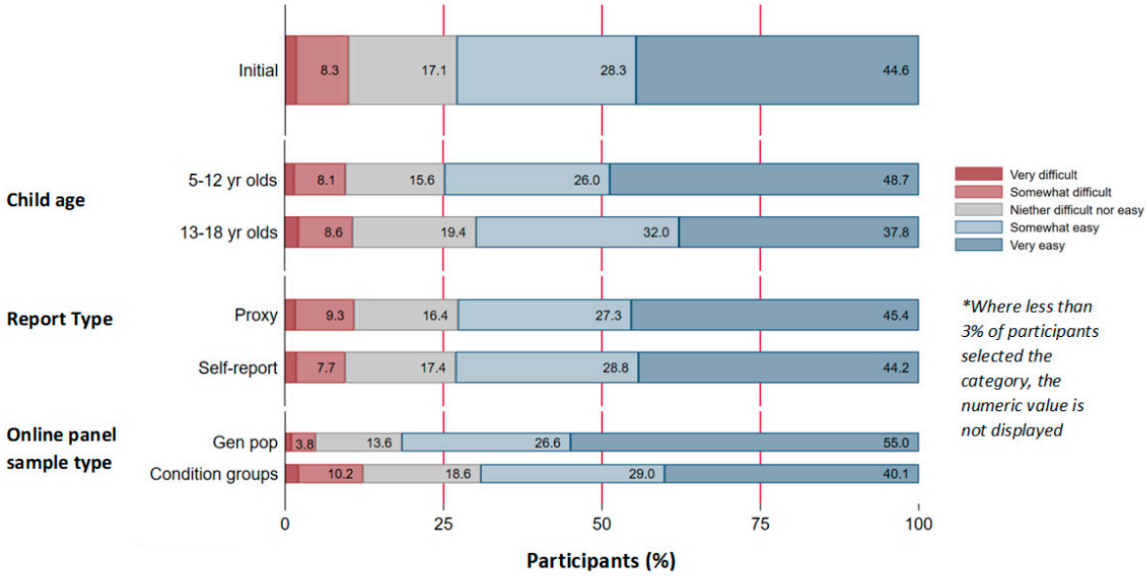

Abbreviations: Gen pop, general population; Initial, initial survey responses.

Figure S1g. PROMIS-25 self-reported difficulty by child age, report type, and online panel sample type.

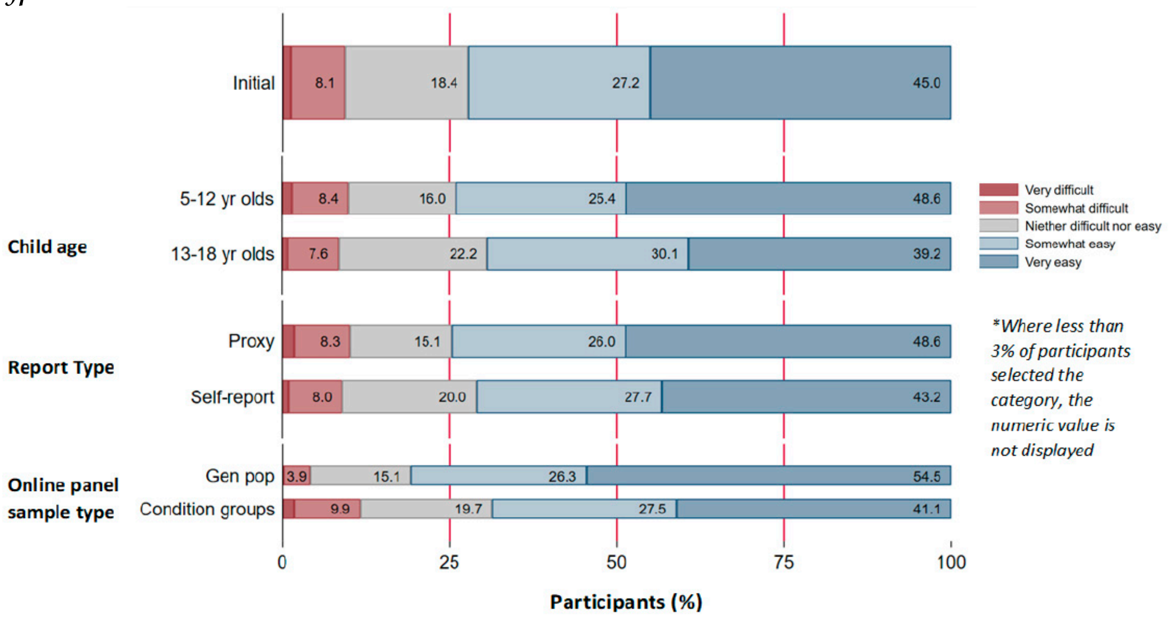

Abbreviations: Gen pop, general population; Initial, initial survey responses.
